# *Salvia miltiorrhiza* in Treating Cardiovascular Diseases: A Review on Its Pharmacological and Clinical Applications

**DOI:** 10.3389/fphar.2019.00753

**Published:** 2019-07-05

**Authors:** Jie Ren, Li Fu, Shivraj Hariram Nile, Jun Zhang, Guoyin Kai

**Affiliations:** ^1^Institute of Plant Biotechnology, School of Life Sciences, Shanghai Normal University, Shanghai, China; ^2^Laboratory of Medicinal Plant Biotechnology, College of Pharmaceutical Science, Zhejiang Chinese Medical University, Hangzhou, China

**Keywords:** cardiovascular diseases, *Salvia miltiorrhiza*, antioxidative, atherosclerosis, endothelial protective, myocardial infarction

## Abstract

Bioactive chemical constitutes from the root of *Salvia miltiorrhiza* classified in two major groups, viz., liposoluble tanshinones and water-soluble phenolics. Tanshinone IIA is a major lipid-soluble compound having promising health benefits. The *in vivo* and *in vitro* studies showed that the tanshinone IIA and salvianolate have a wide range of cardiovascular and other pharmacological effects, including antioxidative, anti-inflammatory, endothelial protective, myocardial protective, anticoagulation, vasodilation, and anti-atherosclerosis, as well as significantly help to reduce proliferation and migration of vascular smooth muscle cells. In addition, some of the clinical studies reported that the *S. miltiorrhiza* preparations in combination with Western medicine were more effective for treatment of various cardiovascular diseases including angina pectoris, myocardial infarction, hypertension, hyperlipidemia, and pulmonary heart diseases. In this review, we demonstrated the potential applications of *S. miltiorrhiza*, including pharmacological effects of salvianolate, tanshinone IIA, and its water-soluble derivative, like sodium tanshinone IIA sulfonate. Moreover, we also provided details about the clinical applications of *S. miltiorrhiza* preparations in controlling the cardiovascular diseases.

## Introduction

Cardiovascular diseases (CVDs) cause nearly one third of all deaths in human beings worldwide (Wong, 2014). Coronary atherosclerotic heart disease, also known as CHD, is the most common type of CVD and one of the deadly diseases among humans (Gao et al., 2017; Liu et al., 2018). The mortality rate of CHD is the highest among all CVDs, accounting for about 40% of all CVD-related deaths (Rezaei-Hachesu et al., 2017; Zhu et al., 2018). Numerous studies have shown that diabetes, hypertension, dyslipidemias, abdominal obesity, unhealthy diet, smoking, and psychosocial stress are major risk factors for CHD (Chow et al., 2007; Gupta et al., 2016; Ajith and Jayakumar, 2018). CHD events were defined as hospitalization for unstable angina pectoris (UAP), myocardial infarction (MI), percutaneous coronary intervention (PCI) or coronary artery bypass grafting, and cardiovascular death (Makino et al., 2015). A number of multicenter randomized clinical trials have been conducted and provided more evidence for the treatment of CVDs by traditional Chinese medicine (TCM) (Chen et al., 2006; Lu et al., 2008; Li et al., 2013; Yu et al., 2018).

**Figure 1:**
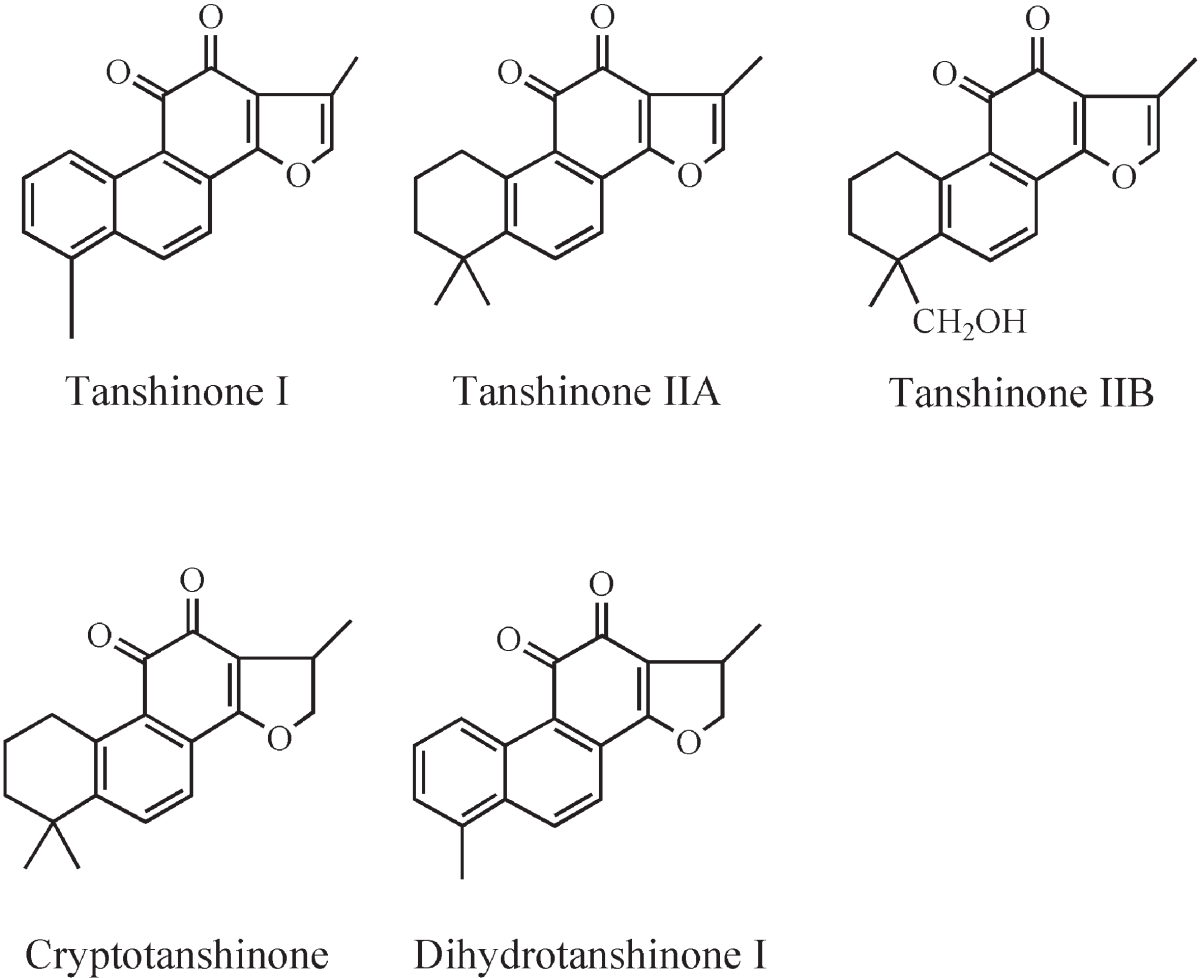
Chemical structures of major tanshinones.

**Figure 2:**
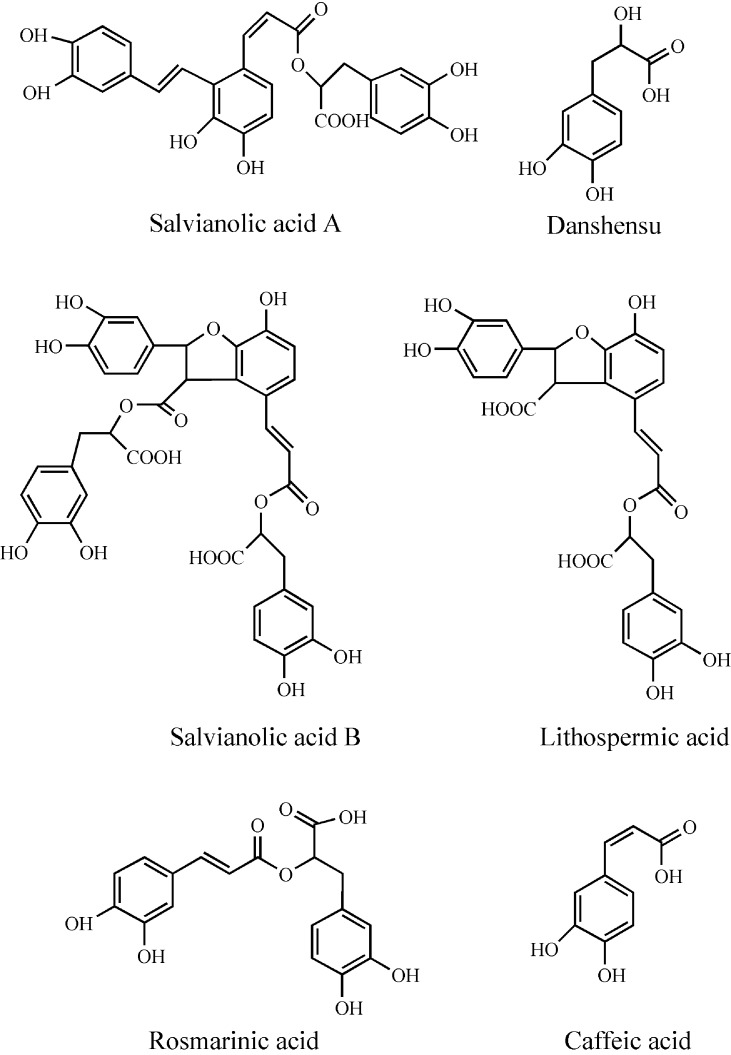
Chemical structures of major salvianolic acids.

**Figure 3:**
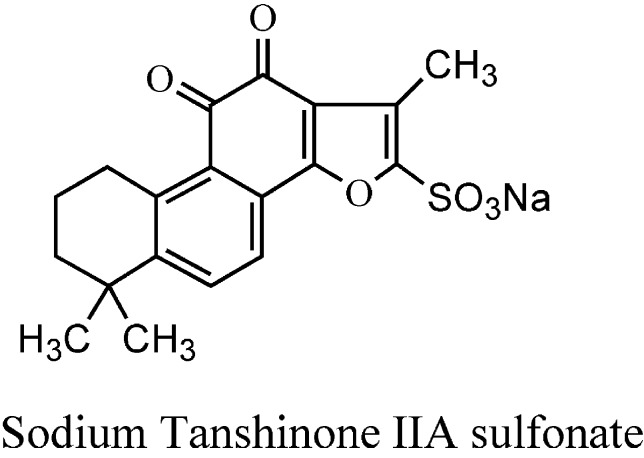
Chemical structures of sodium tanshinone IIA sulfonate.

*Salvia miltiorrhiza* Bunge (SM), known as Danshen, belongs to the family *Labiatae* and is widely used in TCM as a traditional natural medicine in clinics for several decades in various parts of China (Zhou et al., 2017; Cao et al., 2018). Danshen has curative effect alone or in combination with other TCM groups. SM is used to treat malignant tumors, neurological, metabolic disorders, lung diseases, CVDs, inflammatory diseases, gynecological diseases, liver diseases, and renal diseases (Cao et al., 2012; Chen et al., 2013; Yang et al., 2013; Qiang et al., 2015). The chemical constituents from the root extract of SM are divided into two categories: liposoluble tanshinones and water-soluble phenolics (Deng et al., 2019; Huang et al., 2019b; Sun et al., 2019), most of which have been identified and purified using various chromatographic and spectroscopic methods (Zhou et al., 2006). SM contains more than 40 lipophilic constituents and 50 hydrophilic constituents (Zhang et al., 2012; Fang et al., 2018); which are mainly extracted as tanshinone I, tanshinone IIA (TsIIA), tanshinone IIB, cryptotanshinone, and dihydrotanshinone I (**Figure 1**) (Ma et al., 2015). The major phenolic acid constituents among salvianolic acids are salvianolic acid A (Sal A), salvianolic acid B (Sal B), lithospermic acid, danshensu, caffeic acid, and rosmarinic acid (**Figure 2**) (Ho and Hong, 2011; Ma et al., 2013; Shi et al., 2019). Salvianolates are the main water-soluble bioactive compounds extracted from SM and are composed of Sal B, rosmarinic acid, and lithospermic acid, which are widely used in the treatment of CHD (Qi et al., 2017; Qiu et al., 2018). Tanshinones from SM are more effective against treatment of CVDs and cerebrovascular diseases, including atherosclerosis (AS), MI, cardiac hypertrophy (Gao et al., 2012), myocardial ischemia reperfusion (I/R) (Li et al., 2016), and chronic heart failure (HF) (He et al., 2016). The most studied class of tanshinones is TsIIA, which is one of the major bioactive components of SM having less water solubility compared with other tanshinones (Zhu et al., 2017). Sodium TsIIA sulfonate (STS) (**Figure 3**) is a water-soluble derivative of TsIIA, which has been widely used in China for the treatment of CVDs safely and effectively, including MI and angina pectoris (AP) (Zhang et al., 2014a; Mao et al., 2015; Zhu et al., 2017). Previous studies provided information on SM and its bioactive constituents for their various pharmacological activities, so in this review, we outlined the cardiovascular protective effects of TsIIA and STS, providing details about therapeutic mechanisms of CVDs, as well as the application of SM in clinical CVDs.

## Methods

The Gene Cloud of Biotechnology Information (GCBI) and PubMed databases were used to search for antioxidative, anti-inflammatory, endothelial protective, ischemia/reperfusion, myocardial, anticoagulation, vasodilating, smooth muscle cell, anti-AS, and tanshinones, respectively. The literature data related to the pharmacological effects of TsIIA and STS were manually screened out. Furthermore, the GCBI and PubMed databases were used to search for Danshen, *Salvia miltiorrhiza*, tanshinones, TsIIA, sodium TsIIA sulfonate, salvianolate, and CVDs, respectively.

## The Pharmacological Effects of TsIIA, Sodium TsIIA Sulfonate, and Salvianolate

**Figure 4:**
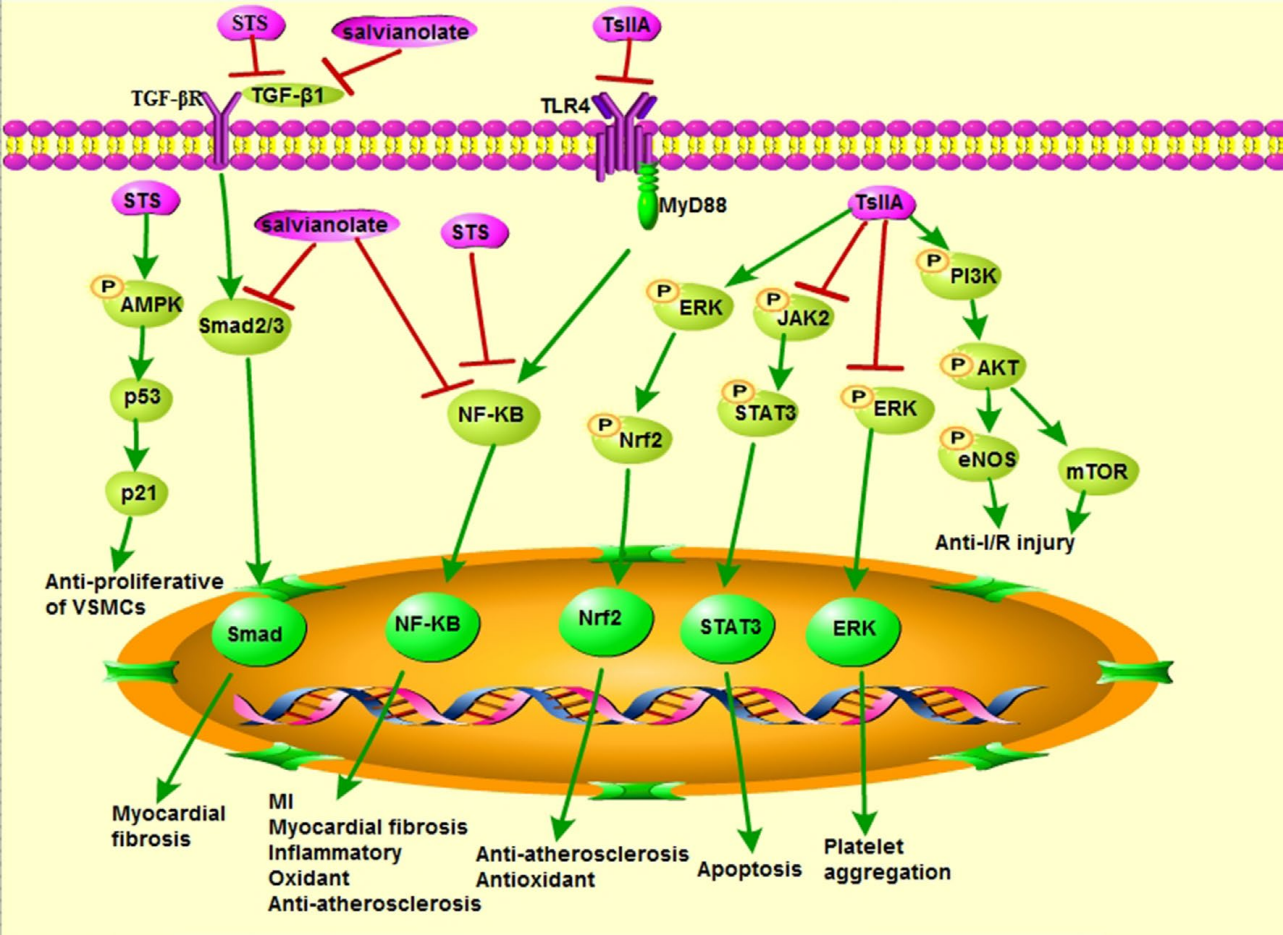
Cardiovascular pharmacological effects of TsIIA, STS, and salvianolate (modified based on Li et al., 2018).

Recent research has demonstrated that TsIIA or STS or salvianolate has numerous cardioprotective effects, including antioxidative (Fei et al., 2013; Xuan et al., 2017), inhibition of apoptosis (Chen et al., 2017c; Yue et al., 2017b), anti-inflammatory (Meng et al., 2014; Feng et al., 2016), anti-cardiac fibrosis (Wu et al., 2018), anti-cardiac hypertrophy (Feng et al., 2017), anticoagulation (Maione et al., 2014), anti-AS (Liu et al., 2014b; Meng et al., 2014), and vasodilating (Li et al., 2015b); also reduction of macrophage derived foam cell formation (Liu et al., 2014b), inhibition of proliferation, and migration of vascular smooth muscle cells (VSMCs) (Wang et al., 2013a; Fang et al., 2018) (**Figure 4**). Therefore, TsIIA and STS can be used as a promising candidate for treating CVDs (**Tables 1** and **2**).

**Table 1:** *In vitro* cardiovascular effects of TsIIA, STS and Salvianolate.

Effects	TsIIA/STS	Cell system	Targets	References
Anti-oxidative	TsIIA	H9c2	Nrf2	(Guo et al., 2018)
Anti-oxidative	STS	H9c2	Cell cycle arrest, oxidative stress, apoptosis	(Zhang et al., 2017b)
Endothelial protective	TsIIA	HUVECs	Multiple ways of post-transcriptional mechanism	(Li et al., 2015b)
Endothelial protective	STS	HUVECs	PI3K/AKT/eNOS pathway	(Cheng et al., 2017)
Endothelial protective	Salvianolate	Primary cardiomyocyte	Smad2/3, TGFβ1	(Fei et al., 2013)
Protective effect against I/R	TsIIA	Myocardial cell	PI3K/Akt/mTOR signaling Pathway	(Li et al., 2016)
Myocardial protective	TsIIA	MMEC	Apoptosis, JAK2/STAT3 signaling pathway	(Cui et al., 2016)
Myocardial protective	Salvianolate	H9c2	Apoptosis, ATP level	(Yue et al., 2017b)
Anti-cardiac hypertrophy	TsIIA	H9c2	IGF-2R pathway	(Chen et al., 2017d)
Anti-cardiac fibrosis	STS	Human atrial fibroblasts	Oxidative stress, TGF-β1 signaling pathway	(Chen et al., 2018)
Anti-cardiac remodeling	TsIIA	H9c2	β-catenin and IGF-2R pathways, apoptosis	(Chen et al., 2017c)
Anti-cardiac remodeling	TsIIA	Human cardiac fibroblasts	ECM remodeling responses	(Mao et al., 2016)
Vasodilating	TsIIA	HUVECs	eNOS, multiple ways of post-transcriptional mechanism	(Li et al., 2015b)
Reduction of VSMCs migration and proliferation	TsIIA	Primary VSMCs	ERK1/2 MAPK signaling pathway	(Lu et al., 2018)
Reduction of VSMCs migration and proliferation	STS	Primary VSMCs	AMPK-p53-p21 signaling, AMPK/NF-κB signaling	(Wu et al., 2014)
Anti-atherosclerosis	STS	HUVECs	Anti-oxidant, anti-inflammation, CLIC1	(Zhu et al., 2017)
Anti-atherosclerosis	TsIIA	EPCs	TNF-α, anti-inflammation	(Wang et al., 2015)
Anti-atherosclerosis	TsIIA	Primary VSMCs, RAW264.7	Apoptosis, anti-inflammation	(Wang et al., 2017)
Anti-atherosclerosis	TsIIA	Human monocyte-derived THP-1	ABCA1/ABCG1, ERK/Nrf2/HO-1	(Liu et al., 2014b)
Anti-atherosclerosis	TsIIA	HUVECs, EPCs	TNF-α, VCAM-1, ICAM-1, IKK/NF-κB signaling pathway	(Chang et al., 2014; Yang et al., 2016a)
Anti-atherosclerosis	TsIIA	Human monocyte-derived DCs	Pro-inflammatory cytokines, atherosclerotic plaque instability	(Li et al., 2014)

**Table 2:** *In vivo* cardiovascular effects of TsIIA, STS, and Salvianolate.

Effects	TsIIA/STS	Animals	Targets	References
Anti-oxidative	TsIIA	Mice	Nrf2	(Guo et al., 2018)
Anti-oxidative	STS	Rat	NF-κB pathway	(Qian et al., 2017)
Anti-inflammatory	Salvianolate	Rat	IL-6, CRP	(Meng et al., 2014)
Endothelial protective	TsIIA	Rat	ET-1, ET_B_ receptors, ET_A_ receptors, eNOS, NO	(Chen et al., 2017b)
Protective effect against I/R	TsIIA	Rat	PI3K/Akt pathway, mPTP	(Yuan et al., 2014)
Protective effect against I/R	TsIIA	Rat	PI3K/Akt/mTOR signaling pathway	(Li et al., 2016)
Protective effect against I/R	TsIIA	Rat	ROS, HMGB1, anti-inflammatory	(Hu et al., 2015)
Protective effect against I/R	STS	Rat	Anti-inflammatory, apoptosis, autophagy	(Pan et al., 2017)
Protective effect against I/R	STS	Rat	Myocardial zymogram, anti-oxidant, HO-1	(Wei et al., 2014)
Protective effect against I/R	Salvianolate	Mice	ERK1/2	(Qi et al., 2017)
Protective effect against I/R	TSI	Rat	NDUFA10, SDHA, Sirt1, Sirt3	(Huang et al., 2019a)
Anti-cardiac hypertrophy	TsIIA	Rat	Cys-C/Wnt signaling pathway	(Feng et al., 2017)
Anti-cardiac fibrosis	TsIIA	Rat	TLR4/MyD88/NF-κB signaling pathway	(Wu et al., 2018)
Anti-cardiac remodeling	TsIIA	Rat	SIRT1 signaling pathway, anti-oxidant, anti-inflammatory	(Feng et al., 2016)
Anticoagulation	TsIIA	Rabbit	TNF-α, hemostatic parameters, liver and renal injuries	(Wu et al., 2012)
Anticoagulation	TsIIA	Mice	ERK2 phosphorylation, blood viscosity, microcirculation	(Maione et al., 2014)
Vasodilating	TsIIA	Rat	eNOS, multiple ways of post-transcriptional mechanism	(Li et al., 2015b)
Vasodilating	STS	Rat	BK_Ca_ channel, Ca^2+^ channel	(Zhang et al., 2018c)
Reduction of VSMCs migration and proliferation	STS	Rat	In a dose-dependent manner	(Wang et al., 2013a)
Anti-atherosclerosis	TsIIA	Rat	Lipid deposition, the distribution of HDL subfractions, intake, and efflux of cholesterol	(Jia et al., 2016)
Anti-atherosclerosis	TsIIA	Mice	Porphyromonas gingivalis, anti-inflammatory	(Xuan et al., 2017)
Anti-atherosclerosis	TsIIA	Mice	Anti-inflammatory, atherosclerotic plaque instability	(Zhao et al., 2016)
Anti-atherosclerosis	STS	Mice	Anti-oxidant, anti-inflammation, CLIC1	(Zhu et al., 2017)
Anti-atherosclerosis	Salvianolate	Rat	IL-6, CRP, regulatory T cell	(Meng et al., 2014)

### Antioxidative Effect

Nuclear factor (erythroid-derived 2)-like 2 (Nrf2) is a central regulator of cellular responses to oxidative stress, which plays a critical role in maintaining normal cardiac function (Guo et al., 2018). Nrf2-dependent antioxidant response mediates the protective effect of TsIIA on doxorubicin (DOX)-induced cardiotoxicity, suggesting that TsIIA may be a promising therapeutic adjuvant that prevents the serious side effects of DOX in the heart (Guo et al., 2018). Differentiation of atrial fibroblasts into myofibroblasts plays a pivotal role in atrial fibrosis (Chen et al., 2018). Studies have shown that STS prevents (angiotensin II) Ang II-induced myofibroblast differentiation through inhibiting oxidative stress and suppressing transforming growth factor-β1 (TGF-β1) signaling pathway in human atrial fibroblasts (Chen et al., 2018). STS may also protect cells from X-ray-induced cell cycle arrest, oxidative stress, and apoptosis during the treatment of radiation-induced cardiovascular damage (Zhang et al., 2017b). In addition, STS acts as an antioxidant for inhibiting hemorrhagic shock (HS)-induced organ failure by inhibiting the nuclear factor kappa B (NF-κB) pathway (Qian et al., 2017). Excessive amounts of reactive oxygen species (ROS) cause irreversible damage to DNA, cell membranes, and other cellular structures by oxidizing proteins, lipids, and nucleic acids (Farias et al., 2017; Zhu et al., 2017). Salvianolate may inhibit the production of ROS and increase the antioxidant capacity of cardiomyocytes (Fei et al., 2013). Salvianolate also improved microvascular reflow by inhibiting oxidative stress and apoptosis (Han et al., 2011).

### Anti-Inflammatory Effect

AS is a chronic inflammatory disease of the arterial wall, which is characterized by progressive lipid accumulation in the aortic intima leading to endothelial cell dysfunction and further destruction of the endothelial barrier and vascular tone (Pang et al., 2019). Its pathogenesis is maladaptive immune response and cholesterol metabolism disorder (Sanz and Fayad, 2008; Chan et al., 2017). Inflammation is dominant in AS and CVDs (Tian et al., 2018). Recent studies showed that TsIIA and its derivatives are able to treat CVDs by decreasing the associated inflammatory responses. TsIIA significantly alleviated transverse aortic constriction (TAC)-induced myocardial remodeling by activating the silent information regulator 1 (SIRT1) signaling pathway, probably because it exerts strong antioxidant and anti-inflammatory activities (Feng et al., 2016). STS reduced the expression of tumor necrosis factor-α (TNF-α), interleukin (IL)-6, chloride intracellular channel 1 (CLIC1), vascular cell adhesion molecule 1 (VCAM-1), and intercellular adhesion molecule 1 (ICAM-1) in the atherosclerotic mice; also the antioxidant and anti-inflammatory properties are mediated by inhibiting the expression of CLIC1 and membrane translocation (Zhu et al., 2017). Salvianolate can reduce serum IL-6 and C-reactive protein (CRP) levels in AS rats in a dose-dependent manner (Meng et al., 2014). In addition, the level of IL-6 in macrophages after salvianolate treatment was also significantly reduced (Stumpf et al., 2013).

### Endothelial Protective Effect

Endothelial nitric oxide synthase (eNOS) is the key enzyme that plays an important role in maintaining the homeostasis of vascular endothelial cells (Li et al., 2015b), as the endothelial cell dysfunction is the basis for the development of various cardiovascular complications of diabetes (Gilbert, 2014). Also, the TsIIA inhibited the decrease of eNOS expression and the generation of nitric oxide (NO) induced by high glucose, which exerted this effect through a variety of post-transcriptional mechanisms (Li et al., 2015b). TsIIA plays a protective role by inhibiting strain-induced endothelin-1 (ET-1) expression, increasing the endothelin type B (ET_B_) receptors, reducing the ET_A_ receptors, upregulating eNOS, and increasing the formation of NO during chronic intermittent hypoxia (CIH)-induced endothelial dysfunction (Chen et al., 2017b). Furthermore, STS has multiple functions in vascular endothelial cells; it inhibits the apoptosis of human umbilical vein endothelial cells (HUVECs) induced by heat stress through phosphoinositide-3-kinase (PI3K)/protein kinase B (AKT)/eNOS signaling pathway (Cheng et al., 2017). Oxygen-free radicals impair NO-mediated coronary vasorelaxation affecting basal and agonist-induced NO release and may lead to endothelial dysfunction (Paolocci et al., 2001). Salvianolate inhibited ROS production by downregulating of transcription factors Smad2 and Smad3 (Smad2/3) and TGF-β1 expression, but high concentrations of salvianolate caused cytotoxicity in mouse cardiomyocytes (Fei et al., 2013). Sal A and Sal B protect HUVECs (Yang et al., 2012a) and human aortic endothelial cells (HAECs) (Yang et al., 2011) from damage and improve blood–brain barrier dysfunction (Yang et al., 2016b) by attenuating the production of ROS.

### Protective Effect Against Ischemia/Reperfusion

I/R injury is considered to be the main cause of CHD, which is characterized by aggravation of functional damage, accelerated myocardial cell death, and arrhythmia (Kong et al., 2016; Yu et al., 2016). Studies have demonstrated that the PI3K/Akt pathway is involved in the cardioprotective effects provided by pharmacological pre- and post-conditioning by inhibiting mitochondrial permeability transition pore (mPTP) opening (Tsang et al., 2004; Bopassa et al., 2006; Yuan et al., 2014). Compared with the I/R model group, the group treated with TsIIA (10 mg/kg, IV) prior to reperfusion decreased myocardial infarct size, elevated levels of phosphor-Akt and phosphor-eNOS, and attenuated mitochondrial permeability transition (Yuan et al., 2014). Therefore, pharmacological post conditioning with TsIIA can protect the myocardium from I/R injury by activating PI3K/AKT-eNOS pathway, and the blockage of mPTP opening may be involved in the cardioprotective effect (Yuan et al., 2014). TsIIA also activated the PI3K/AKT/mammalian target of rapamycin (mTOR) signaling pathway to attenuate myocardial I/R injury in rats (Li et al., 2016). Furthermore, TsIIA may inhibit the increased ROS formation caused by myocardial I/R, reduce the expression of the high mobility group box B1 protein (HMGB1), and inhibit inflammation reaction in the myocardial tissue (Hu et al., 2015). Moreover, STS improved I/R-induced myocardial damage by reducing inflammation and apoptosis, enhancing autophagy (Pan et al., 2017). Changes in serum myocardial zymograms (such as creatine kinase-MB, aspartate transaminase, lactate dehydrogenase) can be used as indicators to determine the alterations of membrane integrity and degree of myocardial injury (Panda and Naik, 2008; Wei et al., 2014). STS reduced some consequences of myocardial ischemia, including cardiac antioxidant status, serum myocardial zymogram, microstructural disorders, and cardiac function (Wei et al., 2014). The optimal treatment time window for STS treatment of myocardial I/R injury appears to be within 2 h after reperfusion (Wei et al., 2014). Salvianolate can reduce myocardial I/R injury in rats by reducing mitochondrial DNA oxidative damage, protecting mitochondrial function, and inhibiting cardiomyocyte apoptosis (Yue et al., 2017a). It also can reduce myocardial I/R injury in mice, which involves the extracellular signal-regulated kinase (ERK)1/2 signaling pathway but not the PI3K signaling pathway (Qi et al., 2017). In addition, total salvianolic acid injection (TSI) attenuated I/R-induced myocardial damage by inhibiting oxidative stress, which is related to the activation of Nicotinamide adenine dinucleotide dehydrogenase [ubiquinone] 1 alpha subcomplex 10 (NDUFA10) and succinate dehydrogenase complex, subunit A, and flavoprotein variant (SDHA) by upregulating Sirtuin1 (Sirt1) and Sirtuin3 (Sirt3) (Huang et al., 2019a).

### The Myocardial Protective Effect

When myocardial cells undergo pathological injury, such as hypoxia injury, I/R injury, cardiac surgery, and diabetic injury, the pathological process can evolve from initial cell edema to degeneration and necrosis of myocardial hypertrophy and fibrosis (Gao et al., 2017). Under the condition of hypoxia/reoxygenation (H/R) injury in rats, TsIIA may alleviate myocardial microvascular endothelial cell (MMEC) apoptosis through inhibiting the Janus kinase 2 (JAK2)/signal transducer and activator of transcription 3 (STAT3) signaling pathway and regulating the expressions of tumor suppressor p53, B-cell lymphoma-2 (Bcl-2), Bcl-2-associated X protein (Bax), and caspase-3 (Cui et al., 2016). Salvianolate may reduce oxidative damage of mitochondrial DNA, protect mitochondrial function, and inhibit cardiomyocyte apoptosis, thereby reducing H/R injury of cardiomyocytes (Yue et al., 2017b). In case of cardiac hypertrophy in spontaneously hypertensive rats (SHRs), it was demonstrated that TsIIA may inhibit cardiac hypertrophy by inhibiting the cystatin c (Cys-C)/Wingless (Wnt) signaling pathway (Feng et al., 2017). TsIIA also attenuates the Ang II-induced pathological hypertrophy by estrogen receptors (ERs) in H9c2 cardiomyoblast cells (Chen et al., 2017d). Furthermore, it was reported that TsIIA attenuated MI and cardiac fibrosis in rats by inhibiting Toll-like receptor 4 (TLR4)/myeloid differentiation primary response 88 (MyD88)/NF-κB signaling pathway (Wu et al., 2018). Sal B can alleviate myocardial fibrosis by reducing Ang II-induced NF-κB activation *in vitro*, thus reversing the process of myocardial fibrosis (Wang et al., 2018a).

Ventricular compensation and secondary pathophysiological responses are accompanied by a series of ventricular myocardial damage and ventricular remodeling for the pathological repair (Gao et al., 2017). A study proved that TsIIA attenuated β-catenin and insulin-like growth factor-II receptor (IGF-2R) pathways, decreased subsequent apoptosis and cardiac remodeling, and promoted survival in H9c2 cardiomyoblasts (Chen et al., 2017c). There is evidence that TsIIA significantly ameliorated myocardial remodeling induced by pressure overload through SIRT1 signaling pathway in TAC rats (Feng et al., 2016). TsIIA is important for the treatment of pathological cardiac remodeling; it can inhibit Ang II-induced extracellular matrix (ECM) remodeling responses in human cardiac fibroblasts (Mao et al., 2016).

### Anticoagulation Effect

Under physiological conditions, coagulation and hemostasis system in the human body are mutually restricted by anticoagulation and fibrinolytic system, but they are in a state of dynamic balance. In pathological conditions, no matter which system is abnormal, it can cause bleeding or thrombosis. There is evidence that TsIIA exerts a significant protective effect against lipopolysaccharide (LPS)-induced disseminated intravascular coagulation (DIC) in rabbits. TsIIA also can improve organ injury and reduce the lethal effects of LPS-treated animals (Wu et al., 2012). TsIIA inhibits platelet aggregation induced by adenosine diphosphate (ADP) and collagen via regulating the acetylation of tubulin and inhibiting ERK2 phosphorylation. Therefore, the compound from SM is a promising drug that can improve blood viscosity and microcirculation to prevent CVDs (Maione et al., 2014).

### Vasorelaxant Effect

The vasculature plays a vital role in maintaining blood pressure and providing adequate hemoperfusion based on dynamic physical conditions (Gao et al., 2017). Impaired endothelium-dependent vasodilation has been thought to play a major role in the development of cardiovascular complications of diabetes (Li et al., 2015b). It was demonstrated that TsIIA may improve impaired endothelium-dependent vasodilation induced by diabetes via enhancing eNOS expression and activity (Li et al., 2015b), and this effect was initiated by a variety of mechanisms at the post-transcriptional level of eNOS, including regulation of eNOS mRNA and protein stability, coupling, and serine 1177 phosphorylation (Li et al., 2015b). Furthermore, STS also exerts vasodilation effect, which not only activated large conductance Ca^2+^-activated K^+^ (BK_Ca_) channel but also blocked Ca^2+^ channel and inhibited Ca^2+^ influx in the VSMCs of rats (Zhang et al., 2018c). Danshen water-soluble extract and Sal B exert their vasorelaxant effects by inhibiting Ca^2+^ influx in VSMCs, and the opening of K^+^ channels has minor contribution to their effects, but does not involve endothelium-dependent mechanism (Lam et al., 2006). The vasodilatation of Sal B depends, at least in part, on NO and its vasodilation associated NO-guanylate cyclase (GC)-cyclic guanosine 3′,5′-monophosphate (cGMP) signals (Shou et al., 2012).

### Reduction of Smooth Muscle Cell Migration and Proliferation

VSMCs play a major role in the pathogenesis of diabetic vascular disease; TsIIA treatment significantly attenuated advanced glycation end products (AGEs)-induced proliferation and migration of VSMCs by inhibiting ERK1/2 mitogen-activated protein kinase (MAPK) signaling pathway (Lu et al., 2018). Therefore, TsIIA may be a potential candidate for the prevention of diabetic AS (Lu et al., 2018). Furthermore, STS may inhibit high glucose-induced VSMCs proliferation and migration via AMP-activated protein kinase (AMPK) activation; it exerts anti-proliferative effect through activating the AMPK-p53-p21 signaling pathway and anti-migration effect by inhibiting AMPK/NF-κB (Wu et al., 2014). These facts prove that STS can be used to prevent AS and restenosis after PCI (Wu et al., 2014), and it helps to prevent the development of pulmonary arterial hypertension (PAH) by inhibiting the expression of canonical transient receptor potential (TRPC)1 and TRPC6, leading to normalized basal intracellular Ca^2+^ concentration ([Ca^2+^]_i_) and attenuated the proliferation and migration of pulmonary arterial smooth muscle cells (PASMCs) (Wang et al., 2013a). Sal B, a major component of salvianolate, inhibited stromal cell-derived factor-1α (SDF-1α)-stimulated cell proliferation and VSMCs migration by inhibiting C-X-C chemokine receptor type 4 (CXCR4) receptor (Pan et al., 2012).

### Anti-AS Effect

AS is an inflammatory disease that causes hardening and thickening of the arterial wall and the formation of plaques, including mesenchymal cells, immune cells, lipids, and ECM (Souilhol et al., 2018). Lesions mainly occur in large and medium elastic muscle arteries, which may cause ischemia of the brain, heart, and extremities, or stroke (Fenyo and Gafencu, 2013). The lesions may exist throughout the entire life of the person through circulation of oxidized low-density lipoprotein (ox-LDL) and other pro-atherosclerotic risk factors (such as hyperhomocysteinemia, hyperglycemia) that trigger multiple major pro-atherogenic events, such as endothelial dysfunction, proliferation, and migration of VSMCs, macrophage-derived foam cell formation, T lymphocyte infiltration, and thrombosis (Fenyo and Gafencu, 2013; Xu et al., 2013; Xu et al., 2014; Tabas et al., 2015; Fang et al., 2018).

TsIIA can affect high-density lipoprotein (HDL) subtractions distribution and the intake and efflux of cholesterol (Jia et al., 2016); inhibit endothelial progenitor cell (EPC) injury (Wang et al., 2015), apoptosis of VSMCs (Wang et al., 2017), proliferation and migration of macrophages (Wang et al., 2017), foam cell formation (Liu et al., 2014b), and vascular inflammation (Yang et al., 2016a; Wang et al., 2017; Xuan et al., 2017); and enhance vulnerable plaque stability (Li et al., 2015a; Zhao et al., 2016). These effects can inhibit the progression of AS. TNF-α impaired EPCs’ proliferation, migration, adhesion, and vasculogenesis ability *in vitro* and promoted EPC secretion of inflammatory cytokines, including IL-6, soluble form of CD40 ligand (sCD40L), and monocyte chemoattractant protein 1 (MCP-1), but TsIIA can reverse these effects (Wang et al., 2015). TsIIA attenuated ox-LDL-induced apoptosis of VSMCs, inhibited ox-LDL-induced proliferation and migration of RAW264.7 cells, and inhibited upregulation of TNF-α, IL-1β, IL-6, and MCP-1 in RAW264.7 cells treated with ox-LDL (Wang et al., 2017); TsIIA reduced scavenger receptor class A (SR-A)-mediated ox-LDL uptake by inhibiting activator protein-1 and increased ATP-binding cassette transporter A1 (ABCA1)/ABCG1-mediated cholesterol efflux through the ERK/Nrf2/heme oxygenase-1 (HO-1) pathway, ultimately leading to reduced cholesterol accumulation in foam cells and atherosclerotic plaques (Liu et al., 2014b). In HUVECs (Chang et al., 2014) and EPCs (Yang et al., 2016a), the TNF-α-induced VCAM-1 and ICAM-1 expression is regulated by inhibiting TNF-α-induced nuclear translocation of NF-κB and activation of IκB kinases (IKK)/NF-κB signaling pathway. TsIIA can also prevent inflammatory responses induced by *Porphyromonas gingivalis* infection in apolipoprotein E knockout mice (ApoE^-/-^) mice, and reduce the expression of inflammatory mediators associated with progression of AS (Xuan et al., 2017). These demonstrated the anti-inflammatory effect of TsIIA in AS. Furthermore, the anti-inflammatory and antioxidant properties of STS in the prevention of AS are mediated by inhibition of CLIC1 expression and membrane translocation (Zhu et al., 2017). TsIIA inhibited dendritic cell (DC) maturation and reduced the expression of pro-inflammatory cytokines while attenuating their ability to stimulate T-cell proliferation and cytokine secretion, which may contribute to the pathophysiological processes involved in atherosclerotic plaque instability (Li et al., 2014). The potential mechanism by which TsIIA stabilized vulnerable plaques in ApoE^-/-^ mice may interfere with AGEs and NF-κB activation, as well as downregulation of downstream inflammatory factors, including ICAM-1, VCAM-1, and matrix-metalloproteinases (MMP)-2, -3, and -9 (Zhao et al., 2016). Salvianolate treatment can dose-dependently reduce AS by reducing the levels of pro-inflammatory cytokines and increasing the number of regulatory T cells (Meng et al., 2014).

## *S. miltiorrhiza* for the Treatment of Clinical Cardiovascular Diseases

TsIIA is currently used in China for the treatment of patients with CHD and ischemic stroke, but TsIIA is not easily absorbed by the intestinal pathway, and then STS injection has been developed to improve the bioavailability of the herbal medicine (Yu et al., 2018). Danshen, Danhong, salvianolate, STS injections, and other SM preparations are widely used in China to treat stable AP (SAP) caused by CHD (Zhang et al., 2018a). Salvianolate injections are composed of water-soluble extract of SM (Han et al., 2011; Li et al., 2019). Danhong injection is a modern patented Chinese medicine extracted from SM and *Flos Carthami* (Zou et al., 2018; Feng et al., 2019). It was approved by the China Food and Drug Administration (FDA) in 2002 (Zou et al., 2018). Compound Danshen dripping pills (CDDP) are a modern Chinese medicine preparation consisting of SM, *Panax notoginseng*, and borneol (Jia et al., 2018). It is the first TCM approved by the American FDA for the treatment of CVDs in Phase II clinical trials (Luo et al., 2013; Zhang et al., 2018b). Therefore, the clinical preparations of SM are mainly divided into three categories: simple monomer preparation, such as STS injection; water-soluble complex, such as salvianolate and Danshen injection; and compound preparation of SM, such as CDDP; also the form of SM preparation includes tablets, injections, capsules, formulations, and drop pills (Liu et al., 2007). This article summarizes the scientific literature that reported the effects of SM on clinical CVDs like CHD, hyperlipidemia, and hypertension (**Table 3**).

**Table 3:** Clinical trials of *S. miltiorrhiza* preparations for controlling cardiovascular diseases.

Diseases	Preparations	Dose	Duration	Cases/controls	Primary outcome measures	References
SAP	Salvianolate injection, Danshen drop pills	200 mg/qd, 30 pills/qd	10 days, 60 days	78/78	SAQ, frequency of AP, angina grade, consumption of short-acting nitrates	(Chen et al., 2017a)
UAP	STS injection	60 mg/qd	4 weeks	46/48	FIB level, DD levels, frequency of AP	(Yan et al., 2009)
NSTEMI	SM	3 g/qd, 1 g each time	One month	26/26	ADMA level	(Zhang et al., 2014b)
Hypertension	Fufang Danshen capsule	1 g, twice-daily	12 weeks	30/25	Systolic blood pressure, pulse rate	(Yang et al., 2012b)
PH	STS	1 g/kg/day	8 weeks	5/-	Exercise capacity, Borg dyspnea score	(Wang et al., 2013b)
Congenital heart defects and PH	SM	200 mg/kg	Before cardiac surgery	10/10	ET-1 response, hemodynamic stability	(Xia et al., 2003)
Hyperlipidemia	CDDP	30 capsules/qd, 10 capsules each time	3 months	38/37	Blood lipid levels	(Zhang et al., 2007)

### SAP and UAP

AP can be divided into SAP and UAP. UAP is a common coronary syndrome between SAP and acute MI, which can easily lead to MI or sudden death (Tan et al., 2018). Chronic SAP accounts for about 50% of all patients with coronary artery disease (CAD) (Chen et al., 2017a). Symptoms of chronic SAP are highly associated with the development of atherosclerotic plaque, which blocks at least one large epicardial coronary artery and triggers an imbalance between myocardial oxygen supply and demand (Chen et al., 2017a).

In a randomized, single-blinded, placebo-controlled, adaptive clinical trial, 156 patients with SAP were randomized into either the placebo (glucose) group or the SM extract (salvianolate injection and Danshen drop pills) group in a 1:1 ratio (Chen et al., 2017a). Participants were treated with glucose or salvianolate injection (200 mg/250 ml 0.9% saline injection, IV drip, qd) for 10 days during hospitalization, followed by the open-label Danshen drop spill (30 pills/day) in the SM extract group for 60 days after discharge (Chen et al., 2017a). Using assessment tools, including the Seattle Angina Questionnaire (SAQ), frequency of AP, angina grade, consumption of short-acting nitrates, and so forth, it was demonstrated that SM extract is beneficial for SAP (Chen et al., 2017a). In addition, previous study reported that five SM-based preparations were effective in the treatment of SAP with clinical improvement rate of 72.4% to 91.6% and electrocardiogram (ECG) improvement rate of 54.5% to 71.6% (Zhang et al., 2018a). The order of five SM-based preparations was as follows: Danhong injection > salvianolate injection > STS injection > Danshen injection of bioactive compounds > Danshen injection (Zhang et al., 2018a).

In another randomized controlled trial (RCT), 100 UAP patients were randomized into two groups that received STS injection (60 mg/250 ml 0.9% sodium chloride injection, qd, 4 weeks) combined with a loading dose of 300 mg aspirin and a maintenance dose of 100 mg of aspirin plus baseline therapy, or 250 ml 0.9% sodium chloride injection (qd, 4 weeks) combined with the same doses of aspirin and baseline therapy (Yan et al., 2009). The severity of AP ameliorated in 94 patients who completed the treatment, with a significant amelioration in total effective rate in the trial groups (Yan et al., 2009). STS can significantly reduce the AP attacks in patients with UAP, which may be associated with decreased levels of fibrinogen (FIB) (Yan et al., 2009). Moreover, in 17 RCTs involving 1,372 patients, the meta-analysis showed that the combination of STS injection and Western medicine for the treatment of UAP significantly improved the total effective rate and the total effective rate of ECG and reduced the level of CRP, FIB, and whole blood high shear viscosity (Tan et al., 2018). In 22 RCTs involving 2,050 patients, the meta-analysis showed that combination of salvianolate injection and Western medicine in the treatment of UAP improved the total effective rate and the total effective rate of ECG, and increased the serum NO lever (Zhang et al., 2016). Therefore, the combined use of STS injection and salvianolate injection was more effective than Western medicine (Zhang et al., 2016; Tan et al., 2018).

### Myocardial Infarction

MI, also known as acute MI (AMI), is the most severe manifestation of CAD, which causes more than 4 million deaths in northern Asia and Europe, and more than 2 to 4 million deaths in the United States (Nichols et al., 2014; Gao et al., 2017; Wang et al., 2018b). Atherosclerotic plaque rupture is the cause of approximately 70% MI (Benjamin et al., 2017). Patients who survive from AMI may subsequently suffer HF, manifested as fibrotic scar tissue, thinning of the ventricular wall, and reduced systolic function (Opie et al., 2006; Wang et al., 2018b).

Fifty-two patients with non-ST elevation MI (NSTEMI) undergoing PCI were randomized into two groups that received the conventional therapy (n = 26) or the conventional therapy plus SM (n = 26, 1 g each time, three times per day for 1 month after PCI) (Zhang et al., 2014b). Elevated levels of asymmetric dimethylarginine (ADMA) in serum are associated with cardiovascular events and are one of the important biomarkers for predicting adverse events and patient mortality after PCI (Lu et al., 2003; Derkacz et al., 2011). The plasma ADMA level in the two groups was significantly decreased at day 30 after PCI with statistical difference, but the reduction in the SM treatment group was more obvious (Zhang et al., 2014b). The improvement of prognosis after the application of SM in patients with PCI may be related to the negative regulation of ADMA by SM (Zhang et al., 2014b). One hundred eight patients with AMI undergoing PCI were randomized into two groups that received the routine treatment (n = 46) or the routine treatment plus intravenous infusion of salvianolate injection (n = 62, 200 mg administered once at 24 h before surgery, once a day after surgery, 1 week) (Zhang et al., 2017a). The changes of oxidative stress indexes, hemodynamic indexes, cardiac function indexes, and related biochemical indicators were analyzed in the two groups at 24 h before surgery and the 8th day after surgery (Zhang et al., 2017a). It was found that salvianolate injection can effectively improve oxidative stress, enhance myocardial perfusion volume, and promote cardiac function recovery in the perioperative period of PCI (Zhang et al., 2017a). In a double-blind RCT, 35 patients with STEMI undergoing PCI were eligible for qi-yin deficiency syndrome, and blood stasis syndromes were randomized into two groups that received Western medicine (n = 18) or Western medicine plus American ginseng and SM preparations (n = 17) for 3 months (Qiu et al., 2009). At the state of dobutamine stress, the left ventricular ejection fraction (LVEF) in the treatment group was higher than that in the control group, and the symptoms of TCM were improved (Qiu et al., 2009). Therefore, TCM treatment can improve the clinical symptoms and quality of life of AMI patients undergoing PCI, and is conducive to myocardial microcirculation (Qiu et al., 2009). A statistical study has shown that the mortality of SM preparation plus conventional care AMI patients is approximately halved compared to conventional care alone (Peto odds ratio, 0.46; 95% confidence interval, 0.28–0.75) (Wu et al., 2008).

### Hypertension

Hypertension is a complex disease involving multiple organ systems, a primary modifiable risk factor for heart disease (Ramirez and Sullivan, 2018), and one of the most common non-communicable diseases in the world, with an increasing incidence rate in developing countries (Gupta et al., 2016; Anupama et al., 2017; Miao et al., 2018). Hypertension is often termed the “silent killer” because many hypertensive patients do not know they have the disease before the onset (Ramirez and Sullivan, 2018). Uncontrolled hypertension causes many complications including but not limited to HF, heart attacks, kidney failure, aneurysms, strokes, and dementia (Ramirez and Sullivan, 2018). The other symptoms include aging (Thawornchaisit et al., 2013), overweight or obesity (Shihab et al., 2012; Tsujimoto et al., 2012), dyslipidemia (Laaksonen et al., 2008), resting heart rate (RHR) (Aladin et al., 2016), hyperuricemia (Krishnan et al., 2007), impaired glucose regulation (Morio et al., 2013; Talaei et al., 2014), and estimated glomerular filtration rate (eGFR) (Takase et al., 2012); these are considered independent risk factors for the development of hypertension (Huang et al., 2018a).

In a double-blind, placebo-controlled, randomized, single-center clinical trial, 55 patients with uncontrolled mild to moderate dose for hypertension were randomized into two groups that received Fufang Danshen capsule (formula mixture, 1,000 mg, twice daily, n = 30) or placebo capsules (n = 25) for 12 weeks (Yang et al., 2012b). The results showed that the Fufang Danshen extract had reduced systolic blood pressure and pulse rate; also it was found that it was well tolerated in patients with hypertension, and no significant difference in adverse effects between the two groups was found (Yang et al., 2012b).

### Pulmonary Heart Disease

Cor pulmonale [pulmonary heart disease (PHD)] is a chronic progressive complicated disease that requires continuous treatment and imposes a huge financial burden on individuals and society (Liu et al., 2014a). PHD is defined as right ventricular failure secondary to pulmonary hypertension (PH), which is mainly caused by various lung diseases, such as chronic obstructive pulmonary disease (COPD) or pulmonary vascular disease (Han et al., 2007; Weitzenblum and Chaouat, 2009; Huang et al., 2018b). PH caused by respiratory system diseases and/or chronic hypoxemia is the main pathological mechanism of chronic PHD (Shujaat et al., 2007; Shi et al., 2015). Antibiotics, diuretics, oxygen therapy, vasodilators, and anticoagulants are currently used medicines for the treatment of PHD; also, some studies have shown that the safety and effectiveness of TCM combined with conventional treatment is useful in the treatment of these diseases (Shi et al., 2015).

The results of many clinical trials have indicated that SM and compound Danshen injection may be alternatives to PHD (Liu et al., 2014a). A systematic review of the efficacy and safety of SM and compound Danshen injection in PHD patients involved 2,715 patients identified in 35 RCTs (Liu et al., 2014a). Meta-analysis used I^2^ test for heterogeneity, and randomized or fixed models were selected based on the heterogeneity of the included studies (Liu et al., 2014a). SM and compound Danshen injection have reached favorable conclusions in reducing blood viscosity, plasma viscosity, hematocrit, and mean pulmonary artery pressure (mPAP) by improving blood partial pressure of oxygen (PaO_2_) (Liu et al., 2014a). In a study enrolled in five hospitalized inpatients, these patients were suffering from various types of serious PH and did not receive the sufficient benefits from sildenafil treatment for at least 3 months (Wang et al., 2013b). After 8 weeks of STS infusion, the patient’s exercise capacity improved, and the Borg dyspnea score was significantly reduced, demonstrating that STS alone or in combination with sildenafil for PH treatment showed significant effect (Wang et al., 2013b). In an RCT, 20 children with congenital heart defects and PH were randomly assigned to two groups that received placebo (n = 10) or SM (200 mg/kg, IV, after anesthesia induction, and at the time of rewarming, n = 10) before cardiac surgery (Xia et al., 2003). The outcome has indicated that SM helps to reduce the ET-1 response and is associated with increased hemodynamic stability after surgery, thereby exerting potent antioxidant therapeutic effect (Xia et al., 2003). Moreover, another clinical trial demonstrated that SM can significantly attenuate lipid peroxide reaction, regulate the imbalance of three antioxidant enzymes [RBC superoxide dismutase (SOD), glutathione peroxidase (GSH-Px), catalase (CAT)], and enhance the body’s defense ability against free radical-induced lipid peroxidation damage (Zhang and Chen, 1994).

### Hyperlipidemia

Hyperlipidemia is a common disease caused by abnormal blood lipid metabolism, which is considered to be a highly independent risk factor for atherosclerotic cardiovascular and cerebrovascular diseases, such as CHD and stroke (Shenghua et al., 2018). Hyperlipidemia is the result of complex interactions between genetic and environmental factors, which can be treated by altering the diet and drugs that regulate lipid metabolism through many mechanisms (Chu et al., 2015). More than 50 TCM formulas have been used to treat hyperlipidemia, of which SM is thought to be beneficial to patients primarily by improving cardiovascular function (Xie et al., 2012). In an RCT, 81 hyperlipidemia patients with phlegm and blood stasis syndrome were randomized into two groups that received CDDP (n = 40) or simvastatin (n = 41) for 3 months (Zhang et al., 2007). The results of this study have shown that CDDP has the effective action for lowering the blood lipid levels without impairing liver function, and its protective liver function may be related to its role in improving antioxidant and reducing inflammation (Zhang et al., 2007).

## Conclusion and Future Perspective

TsIIA, the main bioactive component of SM, has many physiological functions, including antioxidative, anti-inflammatory, endothelial protective, myocardial protective, anticoagulation, vasodilation, anti-AS, and reduction of VSMC proliferation and migration. However, TsIIA has poor oral absorption and low bioavailability. It can be used as a water-soluble derivative of STS, for preparation of new dosage forms, such as microemulsion for injection, microspheres, solid dispersions, liposomes, and nanoparticles. Salvianolate, a major hydrophilic compound of Danshen, has a variety of cardiovascular protective effects, including antioxidative, anti-inflammatory, endothelial protection, myocardial protection, vasodilation, and anti-AS. Both TsIIA and salvianolate have cardioprotective effects with significant differences in their action of mechanism and effect (Wang et al., 2011). For example, tanshinone acts early after ischemic injury, mainly by inhibiting intracellular calcium and cell adhesion pathways, whereas salvianolic acid acts primarily by down-regulating apoptosis (Wang et al., 2011). Some problems remain to be resolved and should be studied by targeting water-soluble and lipid-soluble components of SM having more or less effects on other CVDs, and whether their effects are consistent at different pathological stages and intervention mechanism. By reviewing clinical studies, it has been found that SM preparations have a good application in the treatment of CHD, hypertension, hyperlipidemia, PHD, and other diseases. However, there is a slight difference in Danshen preparation as STS injection has curative effect in the treatment of CHD, AP, PHD, and other diseases; The adjuvant medicine for the treatment of CVDs, compound Danshen injection can improve the symptoms of patients especially when combined with Western medicine; salvianolate injection has the function of promoting blood circulation and collaterals, and is often used for the treatment of cardiovascular and cerebrovascular diseases, such as AP and MI; CDDP is one of the typical representatives of compound Danshen preparation, which can be used to treat hypertension, hyperlipidemia, and other diseases. Therefore, SM plays an important role in the treatment of CVDs and has a better understanding of the pharmacological mechanism of the monomers of its active ingredients. The existing clinical research results can only be used as a partial reference. More rigorous scientific clinical research data are needed to support for the selection of Danshen preparations with effective regime in certain CVDs.

TCM contains a variety of active ingredients, which act on multiple targets in a complex disease network. Medicines exert synergistic effects on each target to intervene in the occurrence and development of the disease, and finally achieve therapeutic effects (Gao et al., 2017). At the same time, it is unclear which ingredients have produced practical effects, which makes the monomer of Chinese herbal medicines a hot spot of concern. Both basic research and clinical observation have made progress, which shows that TCM has huge advantages and prospects, but there are still deficiencies, such as the lack of uniform application standards. The composition and target of SM are more complicated, but with less adverse reactions. It is often used in combination with other medicines, which poses a hidden danger for the adverse reactions with combinations. TCMs may be used as a supplement and alternative to primary and secondary prevention of CVDs, but further rigorous design of RCTs is needed to assess the impact of TCMs on total mortality and major adverse cardiovascular events in patients with CVDs (Hao et al., 2017). Multicenter, large samples, and RCTs are also needed to evaluate the safety and efficacy of Danshen preparations for CVDs.

## Author Contributions

JR and SN reviewed relevant literature and wrote this paper. JR, LF, JZ, and GK revised the manuscript. All the authors listed agreed to the publication of this paper.

## Funding

This work was supported by National Natural Science Fund of China (81522049, 31571735, and 31270007), the “Dawn” Program of Shanghai Education Commission (16SG38), Shanghai Science and Technology Committee Project (17JC1404300, 15430502700), Zhejiang Provincial Ten Thousands Program for Leading Talents of Science and Technology Innovation, Zhejiang Provincial Program for the Cultivation of High-level Innovative Health Talents.

## Conflict of Interest Statement

The authors declare that the research was conducted in the absence of any commercial or financial relationships that could be construed as a potential conflict of interest.
